# An empirical meta-analysis of the life sciences linked open data on the web

**DOI:** 10.1038/s41597-021-00797-y

**Published:** 2021-01-21

**Authors:** Maulik R. Kamdar, Mark A. Musen

**Affiliations:** 1 Center for Biomedical Informatics Research, Stanford University, Stanford, CA USA; 2 Present Address: Elsevier Health Markets, Philadelphia, PA USA

**Keywords:** Data integration, Databases, Databases, Classification and taxonomy

## Abstract

While the biomedical community has published several “open data” sources in the last decade, most researchers still endure severe logistical and technical challenges to discover, query, and integrate heterogeneous data and knowledge from multiple sources. To tackle these challenges, the community has experimented with Semantic Web and linked data technologies to create the Life Sciences Linked Open Data (LSLOD) cloud. In this paper, we extract schemas from more than 80 biomedical linked open data sources into an LSLOD schema graph and conduct an empirical meta-analysis to evaluate the extent of semantic heterogeneity across the LSLOD cloud. We observe that several LSLOD sources exist as stand-alone data sources that are not inter-linked with other sources, use unpublished schemas with minimal reuse or mappings, and have elements that are not useful for data integration from a biomedical perspective. We envision that the LSLOD schema graph and the findings from this research will aid researchers who wish to query and integrate data and knowledge from multiple biomedical sources simultaneously on the Web.

## Introduction

Over the last decade, the biomedical research community has published and made available, on the Web, several sources consisting of biomedical data and knowledge: anonymized medical records^1^, imaging data^2^, sequencing data^3^, biomedical publications^4^, biological assays^5^, chemical compounds and their activities^6^, biological molecules and their characteristics^7^, knowledge encoded in biological pathways^8,9^, animal models^10^, drugs and their protein targets^11^, medical knowledge on organs, symptoms, diseases, and adverse reactions^12^. However, biomedical researchers still face severe logistical and technical challenges to integrate, analyze, and visualize heterogeneous data and knowledge from these diverse and often isolated sources. Researchers need to be aware of the availability and accessibility of these sources on the Web, and need extensive computational skills and knowledge to query and explore them. To systematically consume and integrate the data and knowledge from these sources for use in their analyses, researchers often spend a significant amount of their time dealing with the heterogeneous syntaxes, structures, formats, schemas, and biomedical entity notations used across these sources.

The complexity and time in inter-disciplinary scientific research drastically increase as the biomedical researcher ends up learning multiple systems, configurations, and means to reconcile similar entities across different sources. In most cases, the biomedical researcher just wishes to retrieve relevant data pertaining to their unique criteria or to retrieve answers to specific queries, such as *“What are the medications prescribed to melanoma patients who have a V600E mutation in their BRAF gene?”*, or *“List molecular characteristics of antineoplastic drugs that target Estrogen Receptor α and have molecular weight less than 300 g/mol”*. However, due to the current state of the biomedical data and knowledge landscape with multiple, isolated, heterogeneous sources, the researcher has to hop across several Web portals (e.g., PubChem^5^) and search engines (e.g., PubMed^4^) for these tasks.

Semantic Web and linked data technologies are deemed to be promising solutions to tackle these challenges, and enable toward Web-scale data and knowledge integration and semantic processing in several biomedical domains, such as pharmacology and cancer research^13–17^. Biomedical researchers have been some of the earliest adopters of these technologies, and have used them to drive knowledge management, semantic search, clinical decision support, rule-based decision making, enrichment analysis, data annotation, and data integration^12,16,18–20^. Moreover, these technologies are used to represent data and knowledge stored in isolated, heterogeneous sources on the Web to create a network of linked open data and knowledge graphs, often referred to as the Linked Open Data (LOD) cloud^21^. The LOD cloud was conceived from a vision that a decentralized, distributed, and heterogeneous data space, extending over the traditional Web (i.e., World Wide Web, or Web of Documents), can reveal hidden associations that were not directly observable^22^. The linked open data and knowledge sources, relevant to biomedicine, in the LOD cloud are collectively described as the Life Sciences Linked Open Data (LSLOD) cloud^13^. A diagrammatic representation of the LSLOD cloud can be found as part of the popular Linked Open Data cloud diagram^23^ (https://lod-cloud.net/).

These technologies have matured enough that they are also being widely adopted by several companies, and subsets of the generated knowledge graphs are used in biomedical applications (e.g., Google^24^, IBM^25^, Elsevier^26^, NuMedii^27^, Pfizer^28^). Semantic Web technologies could also be combined with natural language processing-based relation extraction methods to systematically extract scientific relations between biomedical entities and to make them available for querying and consumption through a structured representation of the knowledge graph^26,29^.

Ideally, the LSLOD cloud should serve as an open Web-based scalable infrastructure through which biomedical researchers can query, retrieve, integrate, and analyze data and knowledge from multiple sources, without the requirement on the part of the researchers to download and manually integrate those sources and be concerned with the location, heterogeneous schemas, syntaxes, varying entity notations, representations, or the mappings to reconcile similar concepts, relations, and entities between these sources. However, it is well known that the LSLOD cloud has several shortcomings for this vision to be achieved. Specifically, most biomedical researchers find it difficult to query and integrate data and knowledge from the LSLOD cloud for use in their downstream applications, since the LSLOD cloud faces a set of challenges similar to those that it had set out to solve in the first place^13,30–34^. The rampant semantic heterogeneity across the sources in the LSLOD cloud, due to the heterogeneous schemas, the varying entity notations, the lack of mappings between similar entities, and the lack of reuse of common vocabularies, leads to the question on whether the LSLOD cloud can truly be considered *“linked”*.

In this paper, we present our results on an empirical meta-analysis conducted on more than 80 data and knowledge sources in the LSLOD cloud. The main contributions of this research can be outlined as follows:

1. Using an approach to automatically extract schemas and vocabularies from a set of publicly available LOD endpoints that expose biomedical data and knowledge sources on the Web, we have created an LSLOD schema graph encapsulating content from more than 80 sources.
2. We estimate the extent of reuse of content in LSLOD sources from popular biomedical ontologies and vocabularies, as well as the level of intra-linking and inter-linking across different LSLOD sources.
3. Combining methods of similarity computation and community detection based on word embeddings, we identify communities of similar content across the LSLOD cloud.

We believe that the resources and findings established through this meta-analysis will aid both: (*i)* the biomedical researchers, who wish to query and use data and knowledge from LSLOD cloud in their applications, and (*ii)* the Semantic Web researchers, who can develop methods and tools to increase reuse and reduce semantic heterogeneity across the LSLOD cloud, as well as develop efficient federated querying mechanisms over the LSLOD cloud to handle heterogeneous schemas and diverse entity notations. In subsequent sections of this paper, we provide a brief overview on the Semantic Web technologies and the LSLOD cloud, delve deeper into the different biomedical data and knowledge sources that were used in this research, and provide an overview on the different methods used to extract schemas and vocabularies from publicly available LOD graphs and to evaluate the reuse and similarity of content across the LSLOD cloud. We will present the results from this empirical meta-analysis of the quality, reuse, linking, and heterogeneity across the LSLOD cloud, summarize some of our key findings and observations on the state of biomedical LOD on the Web, and discuss what it means moving forward on the querying and consumption of data and knowledge on the Web in biomedical applications, in a more systematic and integrated fashion.

The LSLOD schema graph and other pertinent data, as well as the results and visualizations from this meta-analysis are made available online at http://onto-apps.stanford.edu/lslodminer. The code used in this research is made available on GitHub (https://github.com/maulikkamdar/LSLODQuery).

## Background and Related Work

### Semantic web technologies and the life sciences linked open data (LSLOD) Cloud

To achieve the goals of a Linked Open Data (LOD) cloud over the Web, the Semantic Web community has developed several standards, languages, and technologies that aim to provide a common framework for data and knowledge representation — linking, sharing, and querying, across application, enterprise, and community boundaries. Semantic Web languages and technologies have been used to represent and link data and knowledge sources from several different fields such as life sciences, geography, economics, media, and statistics, to essentially create a linked network of these sources and to provide a scalable infrastructure for structured querying of multiple heterogeneous sources simultaneously, for Web-scale computation, and for seamless integration of big data.

Some Semantic Web languages and technologies have been standardized and recommended by the World Wide Web Consortium (W3C) and are also widely used by the biomedical community to create the LSLOD cloud. For example, The Resource Description Framework (RDF), a simple, standard triple-based model for data interchange on the Web, is used to represent information resources (e.g., biomedical entities, relations, classes) as linked graphs on the LSLOD cloud^35^. Each resource is uniquely represented using a HTTP Uniform Resource Identifier (URI), so that users can view information pertaining to that resource using a Web browser (i.e., Web-dereferenceable). An example of an HTTP URI from the LOD graph of DrugBank — a knowledge base of drugs and their protein targets^11^ — is http://bio2rdf.org/drugbank:DB00619, where http://bio2rdf.org/drugbank: is considered to be the URI namespace – a declarative region that provides a scope to the identifiers, and DB00619 is the specific identifier of the drug Gleevec. RDF extends the linking structure of the Web by using the URIs to represent relations between two resources. Hence, RDF facilitates integration and discovery of relevant data and knowledge on the Web even if the schemas and syntaxes of the underlying data sources differ. The vocabularies and schemas of biomedical RDF graphs are generally annotated by the elements from the Resource Description Framework Schema (RDFS) language^36^. Similarly, in the last few years, the biomedical community has adopted the Web Ontology Language (OWL) as the consensus knowledge representation language to develop biomedical ontologies^37^. RDFS vocabularies and OWL ontologies enable the modeling of any domain in the world by explicitly describing existing entities, their attributes and the relationships among these entities. These entities and relationships are usually classified in specialization or generalization hierarchies. An ontology also contains logical definitions of its terms, along with additional relations, to facilitate advanced search functionalities^38^. Whereas, RDF is essentially only a triple-based, schema-less modeling language, semantics expressed in RDFS vocabularies and OWL ontologies can be exploited by computer programs, called reasoners, to verify the consistency of assertions in an RDF graph and to generate novel inferences^13^.

After the data and knowledge sources have been published as LOD using the RDF triple-based model, with semantics encoded using the RDFS and OWL languages, the SPARQL Protocol and RDF Query Language (SPARQL) can be used to query across multiple diverse sources using federated architectures^17,39,40^. While this distributed querying approach is inspired from the relational database community, SPARQL query federation architectures leverage the advantages provided by the graphical, uniform syntax, and schema-less nature of RDF to achieve query federation with minimal effort.

### Representative example on use of semantic web technologies in biomedicine

**Fig. 1:**
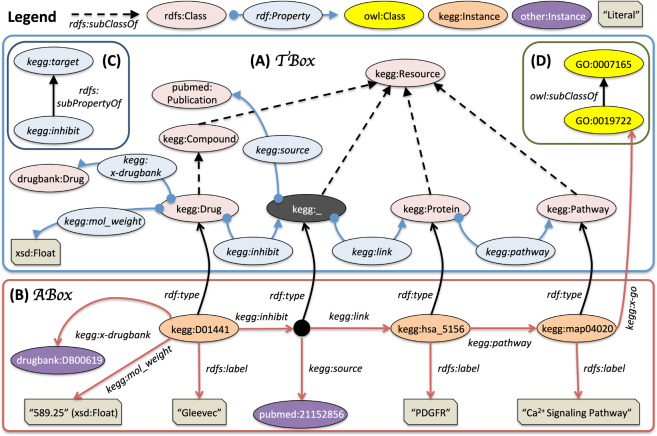
Diagrammatic representation to depict annotated RDF graphs: The KEGG RDF graph whose vocabulary is annotated using RDFS constructs and classes from the Gene Ontology. (**a)** The ${\mathcal{T}}$-Box consists of the terminological component of the KEGG RDF Graph (i.e., the vocabulary). The pink nodes represent the different classes from KEGG and other RDF graph vocabularies (e.g., drugbank:Drug) annotated with rdfs:Class construct. The blue nodes represent the different properties annotated with rdf:Property (with associated domain and range classes). (**b)** The ${\mathcal{A}}$-Box consists of the assertional component of the KEGG RDF graphs, with different individuals (e.g., kegg:D01441) explicitly typed with corresponding classes from ${\mathcal{T}}$-Box. (**c)** The ${\mathcal{R}}$-Box (relational component) consists of the property hierarchy. (**d)** The KEGG individual (pathway Ca^2+^ Signaling Pathway)) may be cross-linked to the corresponding Gene Ontology class, causing a semantic mismatch (i.e., a class is considered equivalent to an individual).

Using Fig. 1, we give a more concrete real-world example on the use of Semantic Web technologies for representing data in the Kyoto Encyclopedia of Genes and Genomes (KEGG) — an integrated data source consisting of several databases, broadly categorized into biological pathways, proteins, and drugs^9^ – and linking the different biomedical entities to similar entities in other biomedical sources, such as DrugBank^11^, PubMed^4^, and Gene Ontology — a unified representation of gene and gene product attributes across all species^19^.

kegg:Drug, kegg:Protein, and kegg:Compound are all RDFS classes in the KEGG vocabulary (Fig. 1a). It should be noted that kegg:Drug is a concise representation for a URI http://bio2rdf.org/kegg:Drug. kegg:Drug is a subclass of kegg:Compound (shown using rdfs:subClassOf). kegg:inhibit and kegg:target are both RDFS properties, with kegg:inhibit being a sub-property of kegg:target (shown using rdfs:subPropertyOf in Fig. 1c). Using a reasoning-enabled query engine, a user who queries for kegg:Compound entities should retrieve all kegg:Drug entities, as well as all non-drug compounds. Similarly, all queries that look for kegg:target relations should retrieve all kegg:inhibit relations, along with non-inhibiting interactions. The domain and range of the properties are also shown using the • and ▸ arrow heads respectively. The KEGG RDF Graph can be aligned to this KEGG RDFS vocabulary, with a class (e.g., kegg:Drug) instantiated with corresponding instances (e.g., kegg:D01441 (Gleevec)) using the rdf:type construct, and properties realized as property assertions between different instances (e.g., kegg:pathway assertion between kegg:hsa_5156 (PDGFR) and kegg:map04020 (Ca^2+^ Signaling Pathway)). These property assertions are shown using red arrows (Fig. 1b).

Figure 1b also shows an example of RDF *reification*, where a *blank node* (represented using a black colored node) is used to capture and represent additional information on the interaction between the drug Gleevec and the receptor protein PDGFR, involved in regulating cell growth and implicated in cancer. The *blank node* links the additional information to a publication with a URI from a PubMed LOD graph. Object properties (e.g., kegg:pathway) generally have rdfs:Class classes as domains and ranges, whereas data properties (e.g., kegg:mol_weight) link an rdfs:Class to a *datatype* (e.g., xsd:Float is the datatype for float numbers).

RDF graphs, annotated using the RDFS language, can be divided into two main components: the terminological component ${\mathcal{T}}$-Box and the assertional component ${\mathcal{A}}$-Box (Fig. 1). The ${\mathcal{T}}$-Box consists of only the axioms pertaining to the vocabulary or schema (e.g. rdfs:Class, rdfs:subClassOf axioms, along with other logical combination constructs). The ${\mathcal{A}}$-Box consists of axioms pertaining to the individuals and their property assertions. RDF graphs (and their associated vocabularies) can be exposed through SPARQL endpoints on the LSLOD cloud for programmatic access.

In general, classes, properties, and instances in RDF graphs may be cross-linked with elements from external ontologies and vocabularies, as well as with entities from other LOD graphs, and making such linkages is often considered a best practice when modeling and publishing datasets on the LOD cloud^41^. For example, kegg:D01441 (Gleevec) of the KEGG RDF graph is linked to a similar entity drugbank:DB00619 of the DrugBank RDF graph, and kegg:map04020 (Ca2 + Signaling Pathway) is mapped to the similar class GO:0019722 in the Gene Ontology **(**Fig. 1b,d**)**.

### Semantic heterogeneity across linked open data graphs

Entity reconciliation and integrated querying are major problems in biomedicine, as there is often no agreement on a unique representation of a given entity or a class of entities^41^. Many biomedical entities are referred to by multiple labels, and the same labels may be used to refer to different entities. For example, similar gene entities could be represented using Ensembl^42^, Entrez Gene^43^, and Hugo Gene Nomenclature Committee (HGNC)^44^ identifiers simultaneously.

One of the key principles for publishing LOD is the use of HTTP Uniform Resource Identifiers (URIs) for entity reconciliation and integrated querying. Each entity (e.g., Lepirudin), or a class (e.g., Drug), should be represented uniquely using one unique URI and other publishers should reuse that URI in their datasets. However, most data publishers create their own URIs by combining the entity IDs from the underlying data sources (e.g., Gleevec DrugBank ID DB00619) with a unique namespace (e.g. http://bio2rdf.org/drugbank:) to represent entities. To resolve the problem of entity reconciliation, efforts such as Bio2RDF^20^ and Linking Open Drug Data^16^ have released guidelines for using cross-reference x-ref attributes rather than using the same URI. Similar entities in different sources should be mapped to each other (e.g., drugbank:DB00619 $\mathop{\leftrightarrow }\limits_{x-ref}$ kegg:D01441 to map Gleevec entities in DrugBank and KEGG), or all similar entities should be mapped to a common terminology (e.g., drugbank:BE0000048 $\mathop{\leftrightarrow }\limits_{x-ref}$ hgnc:3535 $\mathop{\leftrightarrow }\limits_{x-ref}$ kegg:HSA_2147, where the different URIs for protein Prothrombin are mapped to the Hugo Gene Nomenclature Committee (HGNC) identifier) using such x-ref attributes from popular RDFS vocabularies^41^.

Ideally, in the case of RDF datasets, the terminological ${\mathcal{T}}$-Box elements (i.e., classes and properties used in the schemas) should be reused from existing OWL ontologies or RDFS vocabularies. Large repositories of existing RDFS vocabularies are developed, either curated manually (e.g., Linked Open Vocabularies^45^ — a catalog consisting of standardized versions of general RDFS vocabularies that are often used to encode additional semantics for RDF graphs on the LOD cloud) or by exploring the LSLOD cloud through automated crawlers. These repositories can serve as sources for data publishers to reuse elements from existing vocabularies. Moreover, in biomedicine, the classes from existing biomedical ontologies in the BioPortal repository^46^ can also be reused when publishing LOD. RDF instances in the assertional ${\mathcal{A}}$-Box may also be linked to classes from OWL ontologies using equivalence properties, such as x-ref or owl:sameAs as shown in Fig. 1d, where kegg:x-go and kegg:x-drugbank are subproperties of x-ref. However, cases where RDF ‘instances’ are mapped to ‘classes’ in other ontologies or LOD graphs using equivalence properties are often designated as *semantic mismatch* cases. An example of this scenario is depicted in Fig. 1b,d. The pathway Ca2+ Signaling Pathway is represented as a class in the Gene Ontology (GO:0019722), but it is represented as an individual (instance) of the kegg:Pathway class in the KEGG RDF Graph (kegg:map04020). However, these two entities are linked and designated equivalent through the kegg:x-go property.

In some cases, the same relation may be expressed in different RDF graphs using different semantics or graph patterns^13^. For example, the same attribute of molecular weight is expressed in two different LOD sources, DrugBank and KEGG, using drugbank:molecular-weight and kegg:mol_weight data properties respectively. Ideally, the molecular weight attribute assertions should be structured using a uniform property across different sources. This property can originate from any of the LOV vocabularies or biomedical ontologies (e.g. CHEMINF:000216 “average molecular weight descriptor” from the Chemical Information Ontology^47^). As a similar example, drug–protein target interactions are represented using different object properties and graph patterns across DrugBank (e.g., Gleevec $\mathop{\to }\limits_{drug-target}$ PDGFR) and KEGG (e.g., Gleevec $\mathop{\to }\limits_{target}\,\bullet \,\mathop{\to }\limits_{link}$ PDGFR, where the • represents a *blank node* to capture additional information such as the source) RDF graphs. A user may wish to retrieve and reconcile such relations and attributes from both DrugBank and KEGG, as well as multiple other LOD graphs, to gain a complete picture in biomedicine, as unique drug–protein target relations may exist across the sources depending on the nature of their creation, or there may be conflicting information^13,40^. In the current state of the LSLOD cloud, the user must be aware of the underlying semantics and the data model used in these graphs as well as formulate lengthy SPARQL queries, which makes the entire process of information retrieval from the LSLOD cloud non-trivial.

There are several other benefits for reuse or cross-linking between similar entities or classes in different LOD graphs or ontologies^41,48^ — *i)* reduction in data and knowledge engineering costs since the publisher can reuse existing minted URIs or link to existing entity descriptions, *ii)* enabling semantic interoperability among different datasets and applications, and *iii)* querying multiple LOD graphs simultaneously using federated architectures. The lack of reuse or cross-linking, the use of different semantics or graph patterns for modeling data in a given LOD graph, as well as semantic mismatch caused due to linking, are different manifestations of *semantic heterogeneity* across the LSLOD cloud.

### Related research

Previously, we had conducted a study to estimate *term reuse* (i.e., reuse of classes from existing ontologies) and *term overlap* (i.e., similar classes across ontologies which are not reused) across biomedical ontologies stored in the BioPortal repository^48^. We found that ontology developers seldom reuse content from existing biomedical ontologies. However, there is significant overlap across the BioPortal ontologies. The concepts of *term reuse* and *term overlap* also hold true in the context of linked open data (LOD) sources. However, in the biomedical domain, there are several critical differences between ontologies and LOD sources due to the manner through which these sources are created and formalized. Some of these differences include: *i)* the vocabularies and schema for biomedical LOD sources are significantly smaller compared to most biomedical ontologies, *ii)* LOD sources have a much higher number of instances compared to biomedical ontologies and these instances may be reused from external resources, *iii)* LOD vocabularies formalized using the RDFS language may have less rich annotations compared to OWL ontologies (e.g., lack of synonyms and alternate labels), and *iv)* LOD sources, formalized using RDF and RDFS, may use *reification* to capture information at a higher granularity. Hence, the methods to analyze semantic heterogeneity across biomedical ontologies cannot be translated “as is” to analyze semantic heterogeneity across biomedical LOD sources.

A recent study found that the coverage of biomedical ontologies and public vocabularies is not sufficient to structure the entirety of biomedical LOD^49^. Hence, linked data publishers end up creating their own custom vocabularies, and do not publish them in a standardized way for reuse in other projects. Recently, multiple studies have been conducted on the larger Linked Open Data cloud to investigate the availability and licensing of Semantic Web resources, as well as to provide a mechanistic definition of what constitutes a ‘link’ in open knowledge graphs available on the Web^30,33,50^. However, these studies do not particularly focus on the semantic heterogeneity and vocabulary reuse across the LSLOD cloud^51^

We use an Extraction Algorithm to extract and merge the schemas and vocabularies used by LSLOD sources for conducting our meta-analysis. The theory behind the Extraction Algorithm has identical characteristics to linked data profiling algorithms^52–55^. However, the linked data profiling algorithms have, in many cases, only been implemented over some of the popular SPARQL endpoints, do not account for variable SPARQL versions, require the retrieval of all instances and assertions that is often not scalable for LSLOD sources, or may not extend behind minimal schema extraction. Similarly, RDF graph pattern mining algorithms (e.g., evolutionary algorithms^56^) often require exhaustive training samples of (*source*,*target*) pairs which are often not possible to obtain for the LSLOD cloud.

## Methods

### Extracting schemas and vocabularies from the LSLOD cloud

For conducting our meta-analysis of the Life Sciences Linked Open Data (LSLOD) cloud, we have selected a set of LOD projects, SPARQL endpoints, and RDF graphs, by querying the metadata of the projects in the “Life Sciences” section of the popular Linked Open Data cloud diagram (April 2018 version)^23,30^, as well as through a literature review^13^ of articles, documenting popular biomedical LOD projects, available on the PubMed search engine^4^. Furthermore, we established the following criteria for an LSLOD source to be included in the meta-analysis: (*i)* Each LSLOD source must have a functional SPARQL endpoint, (*ii)* For cases when an LSLOD source does not have a functional SPARQL endpoint, the source should be available as RDF data dumps that can be downloaded and stored in a local SPARQL repository, and (*iii)* Each LSLOD source must have at least 1,000 instances under any classification scheme that can be queried through the SPARQL endpoint.

The entire list of the different LOD projects and details about sample RDF Graphs evaluated in this research are shown in Table 1. These RDF graphs include popular biomedical data sources such as DrugBank^11^, Kyoto Encyclopedia of Genes and Genomes (KEGG)^9^, Pharmacogenomics Knowledgebase (PharmGKB)^57^, Comparative Toxicogenomics Database (CTD)^58^, exposed by the Bio2RDF consortium^20^, UniProt^7^, ChEMBL^59^, and Reactome^8^, exposed by the EBI-RDF consortium^60^, and other sources such as The Cancer Genome Atlas (TCGA)^3^, PubChem^5^, National Library of Medicine (NLM) Medical Subject Headings (MeSH), National Bioscience Database Center (NBDC), and WikiPathways^61^. It should be noted that some of these projects (e.g., Bio2RDF^20^) have reprocessed publicly available data and knowledge published by other groups (e.g., DrugBank^11^), whereas in some cases data publishers themselves use Semantic Web technologies to provide access to their proprietary data as LOD (e.g., the European Bioinformatics Institute (EBI) RDF Platform^60^). These RDF graphs contain data and information on several biomedical entities such as drugs, genes, proteins, pathways, diseases, protein–protein interactions, drug–protein interactions, biological assays, and high-throughput sequencing. The SPARQL endpoint locations for these projects are listed at http://onto-apps.stanford.edu/lslodminer.

**Table 1:** Linked Open Data sources queried to generate the ${\mathcal{G}}$_*LSLOD*_ schema graph of the LSLOD cloud.

Linked Data Project	Example RDF Graphs and Descriptions
**Bio2RDF**^20^	Several reprocessed data and knowledge sources such as:
	**DrugBank**^11^ — Drug and drug target information
	**KEGG**^9^ — Biological pathways, proteins, and drugs
	**PharmGKB**^57^ — Protein–drug–disease relations
	**CTD**^58^ — Environmental chemical–protein interactions and pathway–disease relations
**EBI-RDF**^60^	Several proprietary data and knowledge sources such as:
	**Reactome**^8^ — Biochemical reactions and pathways
	**BioSamples**^70^ — Metadata about biological samples
	**ChEMBL**^59^ — Drug-like molecules and activities
**UniProt**^7^	UniProt database on proteins and their characteristics
**MO-LD**^94^	Model Organism databases on different species such as Fly, Mouse, Yeast, Rat, Human and Zebrafish
**NBDC RDF**^95^	Japanese Life Sciences databases on chemicals, structures, sequencing data, pathways, and diseases
**Linked Life Data**^91^	Several reprocessed data and knowledge sources such as:
	**EntrezGene**^43^ — Genes and variants in diseases
	**BioGrid**^96^ — Protein and chemical interactions
	**IntAct**^97^ — Protein–protein interactions
**Linked TCGA**^17^	DNA methylation, gene expression and clinical data of cancer patients from The Cancer Genome Atlas^3^
**NIH PubChem**^5^	Databases on substances, compounds, structures, and biological assays stored in the PubChem repository
**WikiPathways**^61^	Database of biological pathways maintained using a crowd-sourcing architecture
**PathwayCommons**^98^	Data warehouse consisting of several biological pathways and molecular interactions datasets
**NLM MeSH**^86^	Medical Subject Headings used to index biomedical publications in MEDLINE repository^67^
**DisGenet**^99^	Data warehouse on genes and variants associated to diseases
**NextProt**^100^	Data warehouse on protein structures and interactions
**LinkedSPL**^101^	Structured product labels of drugs represented as RDF

The meta-analysis relies on a set of extractors that navigate to each SPARQL endpoint and extract the schemas and vocabularies used in the RDF graphs exposed through those SPARQL endpoints. The extractors encapsulate an Extraction Algorithm that uses a set of SPARQL query templates (Box 1) to extract schemas (e.g., classes, properties, domains, ranges) as well as sample instances of those classes and their property values from the SPARQL endpoints in the LSLOD cloud. A snippet of the extracted information for a given source is shown in Box 2. The input to the Extraction Algorithm is the SPARQL endpoint, whose version of SPARQL can be automatically detected based on the SPARQL queries and keywords supported. The Extraction algorithm not only accounts for different versions of SPARQL supported at the remote SPARQL endpoints, but also uses alternative SPARQL queries if the remote endpoint times out during the extraction phase. The Extraction algorithm is presented in Box 3.

An extractor selects the set of SPARQL query templates for execution against the input SPARQL endpoint, given its version (**Step 2**). The extractor queries the SPARQL endpoint and extracts the named RDF Graphs ${\mathcal{N}}{\mathcal{G}}$ exposed through the SPARQL endpoint (**Step 3, SPARQL Query SQ**_**1**_). For each RDF graph, the extractor extracts the set of classes ${\mathcal{C}}$ and the total number of instances for each class *k*(${\mathcal{C}}$) (**Step 7, SQ**_**2**_). The extractor identifies the properties ${\mathcal{P}}$ for which a specific class serves as the domain for all assertions, and also retrieves the range classes *r*(${\mathcal{P}}$) for object properties, or the datatypes *dt*(${\mathcal{P}}$) for data properties (**Step 12, SQ**_**3**_). The Extraction algorithm also extracts a random sample (without replacement) of 2,000 instances for each class (**Step 9, SQ**_**4**_) and 2,000 property assertions (**Step 14, SQ**_**5**_) for a class–property combination, and generates summary characteristics (e.g.is_categorical, datatype, namespace, median length) of the associated data.

**Fig. 2:**
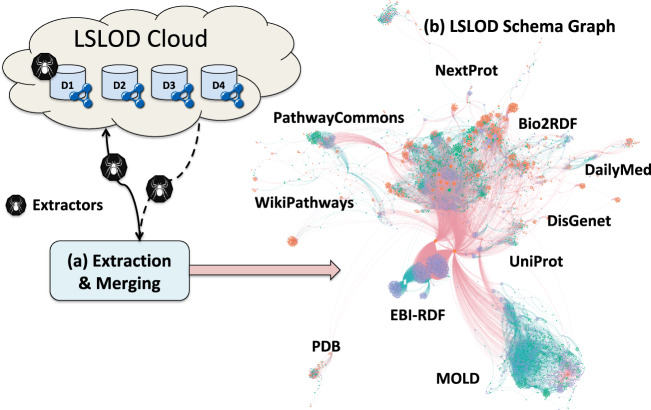
Diagrammatic representation of the Extraction Algorithm: During the extraction phase, automated extractors (represented as black spiders here) query each SPARQL endpoint in the LSLOD cloud and extract the schemas (i.e., classes, object properties, data properties, URI patterns, graph patterns) to generate the LSLOD Schema Graph ${\mathcal{G}}$_*LSLOD*_. A portion of ${\mathcal{G}}$_*LSLOD*_ is visualized here, using a force-directed network, with classes (purple nodes), object properties (green nodes) and data properties (orange nodes) extracted from multiple sources, such as Bio2RDF, EBI-RDF, etc.

Classes, properties, and datatypes, are represented as nodes in the schema graph ${{\mathcal{G}}}_{{\mathcal{T}}}$_*–Box*_ extracted from the SPARQL endpoint by the specific extractor (**Steps 11, 17, 18, 21, 22**). The edges link property nodes to class nodes and datatype nodes depending on the domain and range descriptions of the corresponding property (**Steps 19 and 23**). The nodes and edges are annotated by the count (*k*(${\mathcal{C}}$) and *k*(${\mathcal{P}}$)) and summary characteristics of corresponding instances or property assertions respectively (**Steps 11, 19, 23**). This schema graph is a fragment of the actual ${\mathcal{T}}$-Box of the RDF graph. The ${{\mathcal{G}}}_{{\mathcal{T}}}$_–*Box*_ graphs extracted for all RDF graphs in the LSLOD cloud are merged to generate the schema graph ${\mathcal{G}}$_*LSLOD*_ of the entire LSLOD cloud. A diagrammatic representation of this method and a subset of the merged ${\mathcal{G}}$_*LSLOD*_ schema graph are shown in Fig. 2.

Box 1 SPARQL Query templates to extract schema descriptions from the LSLOD Cloud. ------------------ SPARQL QUERY 1 ------------------

**SELECT DISTINCT** ?g **WHERE** {

                    **GRAPH** ?g {?s ?p ?o}

}

------------------ SPARQL QUERY 2 ------------------

**SELECT** ?Concept (**COUNT** (?x) **AS** ?cCount) **WHERE** {

                    **GRAPH** <GRAPH_URI> {?x **rdf**:type ?Concept}

}**GROUP BY** ?Concept **ORDER BY DESC**(?cCount)

------------------ SPARQL QUERY 3 ------------------

**SELECT DISTINCT** ?p ?c (**COUNT** (?x) **AS**?count)?valType **WHERE** {

                    **GRAPH** <GRAPH_URI> {“?x **rdf**:type <CONCEPT_URI> ;?p ?o.

                    **OPTIONAL** {?o **rdf**:type?c}.

                    **FILTER**(!(?p = ’ **rdf**:type’)).                    

**BIND** (DATATYPE(?o) **AS** ?valType)}

} **GROUP BY** ?p ?c ?valType **ORDER BY DESC**(?count)

------------------ SPARQL QUERY 4 ------------------

**SELECT** ?x **WHERE** {

                    **GRAPH** <GRAPH_URI> {?x **rdf**:type <CONCEPT_URI> }

} **ORDER BY** RAND() **LIMIT** 2000

------------------ SPARQL QUERY 5 ------------------

**SELECT** ?x **WHERE** {

                    **GRAPH** <GRAPH_URI> { ?c **rdf**:type <CONCEPT_URI> ; <PROPERTY_URI> ?x }

} **ORDER BY** RAND() **LIMIT** 2000

Box 2 Information from the Bio2RDF DrugBank RDF Graph extracted by the Extraction Algorithm. Source: DrugBank

Source URI: http://bio2rdf.org/drugbank_resource:bio2rdf.dataset.drugbank.R3

Classes:

              Class: Drug

                          Class URI: http://bio2rdf.org/drugbank_vocabulary:Drug

                          Sample Instances: [’http://bio2rdf.org/drugbank:DB03536’,…]

              Class: Drug-Drug-Interaction

                          Class URI:http://bio2rdf.org/drugbank_vocabulary:Drug-Drug-Interaction

                          …

Properties:

              Object Property: target

                          Property URI: http://bio2rdf.org/drugbank_vocabulary:target

                          Property Realization: drugbank:Drug - > drugbank:target

                                       Domain: Drug, Range: Target

                                       Sample Assertion Values: [’http://bio2rdf.org/drugbank:BE0000059’,…]

              Data Property: molecular-weight

                          Property URI: http://bio2rdf.org/drugbank_vocabulary:molecular-weight

                          Property Realization: drugbank:Enzyme - > drugbank:molecular-weight

                                       Domain: Enzyme, Range: Float

                                       Sample Assertion Values: [52221.099999999999, 56345.0, 57530.0,…]

Box 3 LSLOD Schema Extraction Algorithm.

### Determining vocabulary reuse across LSLOD sources

Estimating the extent of observed vocabulary reuse across different LSLOD sources requires identifying the classes and properties most commonly reused from standard ontologies and vocabularies. For this goal, along with the ${\mathcal{G}}$_*LSLOD*_ schema graph extracted from the LSLOD cloud, we use two different corpora of ontologies and vocabularies:

1. ***BioPortal repository of biomedical ontologies:*** BioPortal repository is the world’s largest open repository of biomedical ontologies^46^. We obtained a dump of 685 distinct biomedical ontologies, serialized using the N-triples format and versioned up to January 1, 2018. This dump did not contain some ontologies that were deprecated or merged with existing ontologies, or added to BioPortal after January 1, 2018. This corpus includes ontologies such as Gene Ontology (GO)^19^, National Cancer Institute Thesaurus (NCIT)^15^, Chemical Entities of Biological Interest (ChEBI) Ontology^6^, and Systematized Nomenclature of Medicine - Clinical Terms (SNOMED CT)^62^, which are widely used in biomedicine for data annotation, knowledge management, and clinical decision support.
2. ***Linked Open Vocabularies (LOV):*** LOV is a catalog of RDFS vocabularies available for reuse with the aim of describing data on the Web^45^. Popular examples of RDFS vocabularies include the W3C Provenance Interchange^63^, Simple Knowledge Organization System (SKOS)^64^, and Schema.org^65^. We used 647 vocabularies that were present in the LOV catalog, as of January 1, 2018. The vocabularies in this catalog generally include concept descriptions (i.e., human-readable labels using the rdfs:label annotation properties).

For each URI in the ${\mathcal{G}}$_*LSLOD*_ schema graph (class, property, or sample instance URI), the *origin* of the URI is determined using a heuristic approach^66^ by converting each URI to lowercase and using *regular expression* filters constructed from namespaces (e.g., ncicb.nci.nih.gov for NCIT^15^) and common identifier patterns.

For each LSLOD source, we compute the following statistics:

1. The number of ontologies and vocabularies reused in the source
2. The percentage of schema elements reused from external LSLOD sources
3. The percentage and distribution of classes whose entities are mapped or linked to other entities in external LSLOD sources (inter-linking)
4. The percentage and distribution of classes whose entities are mapped or linked to entities of other classes in the same LSLOD source (intra-linking)
5. The percentage of entities reused or mapped to external LSLOD sources

Previously, we outlined a method to estimate reuse of classes across multiple biomedical ontologies^48^. We repurposed this method to estimate vocabulary reuse across biomedical LOD sources. We generated an undirected network composed of different URIs from the ${\mathcal{G}}$_*LSLOD*_ schema graph as nodes (i.e., the classes and the properties), and the edges indicating reuse and mapping between the different schema element URIs. “Patterns” of instance URIs are also represented as nodes in this network to check if instances are reused or mapped to classes in biomedical ontologies (i.e., semantic mismatch). We extract connected components from this network and use Eq. 1 to estimate vocabulary reuse.

1 $$Vocabulary\,Reuse=\frac{\sum _{j|{T}_{j}\in \,{{\mathcal{M}}}_{r}}{n}_{j}-k}{N}$$

In the above equation, assume that the network is composed of *k* connected components {${\mathcal{T}}$_0_, ${\mathcal{T}}$_1_, …, ${\mathcal{T}}$_*k*_}, and each component ${\mathcal{T}}$_*j*_ is formed from *n*_*j*_ schema elements (i.e., {*t*_0*j*_, …, *t*_*nj*_}∈ ${\mathcal{T}}$_*j*_). Assume that *N* is the total number of schema element URIs in the network. The number of terms in a component *T*_*j*_ must follow 1 < *n*_*j*_ < *N* (i.e., components with a single term are not allowed). All schema elements in one component exhibit some reuse with respect to each other.

### Detecting communities of related content in the LSLOD cloud

**Fig. 3:**
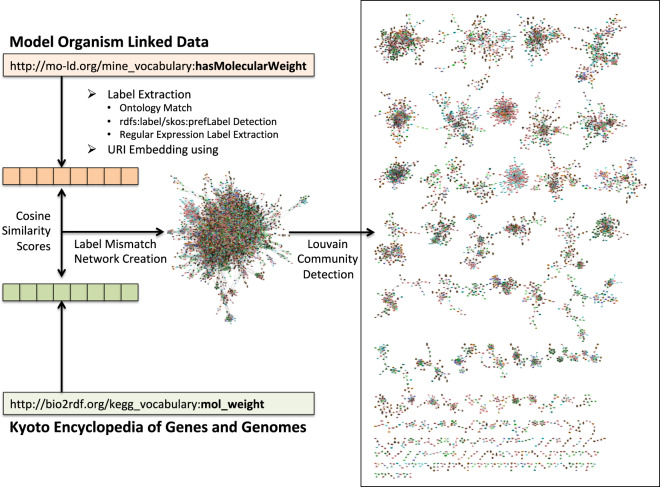
Diagrammatic representation of the algorithm to detect communities of similar content across LSLOD cloud: The labels for two URIs are extracted either (*i)* by matching to a reused ontology, or (*ii)* by using annotation properties such as *rdfs:label* or *skos:prefLabel*, or (*iii)* by using regular expressions on the URI. URI embeddings are generated from the extracted labels, by using a background source of MEDLINE biomedical abstracts. A similarity network is created with nodes representing the different URIs from the ${\mathcal{G}}$_*LSLOD*_ schema graph and edges representing the cosine similarity scores between two URI embeddings. We detect communities in this network using the Louvain method. The trivial example shown here is related to similar “molecular weight” URIs between the MO-LD project and the Bio2RDF KEGG LOD graph.

To detect clusters of related content in the LSLOD cloud, we used a 5-step algorithm that maps similar schema elements (e.g., classes and properties) between two different sources in the LSLOD cloud. A diagrammatic representation of this algorithm is shown in Fig. 3. This algorithm uses the schema elements extracted from the various LSLOD public SPARQL endpoints (see **1**) and included in the ${\mathcal{G}}$_*LSLOD*_ graph. These schema elements may or may not be reused from a common vocabulary or ontology (vocabulary reuse in LSLOD cloud was determined using the methods described in the previous section).

The algorithm uses two sources to generate the mappings between two different schema elements:

1. ***The set of biomedical ontologies and vocabularies that are reused in the LSLOD cloud:*** Biomedical ontologies and vocabularies whose elements are reused across multiple RDF graphs were determined using the previous methods.
2. ***Pre-trained word embedding vectors:*** We use 100-dimensional word embedding vectors pre-trained from a corpus of approximately 30 million biomedical abstracts, stored in the MEDLINE repository^67^, using the GloVe (Global Vectors for Word Representation) method^68^. Word embeddings represent words as high-dimensional numerical vectors based on co-occurrence counts of those words as observed in the abstracts. The pre-trained embedding vectors and inverse document frequency (IDF) statistics for a vocabulary of 2,531,989 words are available under a CC BY 4.0 license at figshare^69^. These word embedding vectors have been used to map metadata fields in the BioSamples repository^70^ to ontology terms in the BioPortal repository^71^, as well as to identify biomedical relations in unstructured text^26^.

### Label extraction

In the first sweep, the algorithm extracts a label for each schema element URI (i.e., class, object property or data property) in the ${\mathcal{G}}$_*LSLOD*_ graph. This is done through three approaches in a sequential flow: [noitemsep]

1. A label is extracted from the values for the rdfs:label^36^ or skos:prefLabel^64^ annotation properties, if the ${\mathcal{G}}$_*LSLOD*_ schema element has already been reused from existing biomedical ontologies and vocabularies (see **Datasets**).
2. A label is extracted by querying the source (i.e., the SPARQL endpoint) of the schema element for rdfs:label or skos:prefLabel annotation assertions.
3. A label is generated using Regular Expression (*RegExp*) filters from the URI of the schema element. Specifically, the filters deal with cases where separators (e.g., “-”, “_”) and camel case (e.g. hasMolecularWeight) is present in the URI. In Fig. 3, the labels “Has Molecular Weight” and “Mol Weight” will be extracted from the two URIs respectively.

### URI embedding generation

We use the pre-trained word embedding vectors to represent each schema element URI in a high dimensional space. Specifically, a URI embedding vector is generated by computing a weighted average of the embedding vectors of the words in the URI label, with the weights being the IDF statistic for each word. A default embedding vector and IDF statistic is created for any word that is not present in the vocabulary. The equation to generate a URI embedding vector is shown below. Here, **x**_(*wi*)_ represents the 100-dimensional word embedding vector, and ${\mathcal{L}}$(*URI*) is the URI label.

2 $${{\bf{x}}}_{(URI)}=\frac{\sum _{{w}_{i}\in {\mathcal{L}}(URI)}id{f}_{({w}_{i})}\ast {{\bf{x}}}_{({w}_{i})}}{\sum _{{w}_{i}\in {\mathcal{L}}(URI)}id{f}_{({w}_{i})}}$$

### Cosine similarity score computation

For any two schema element URIs that are not present in the same source, cosine similarity scores are computed using their URI embedding vectors. The equation to compute the similarity between two elements is presented below.

3 $$Sim(A,B)=\frac{{{\bf{x}}}_{(UR{I}_{A})}\cdot {{\bf{x}}}_{(UR{I}_{B})}}{\left\Vert {{\bf{x}}}_{(UR{I}_{A})}\right\Vert \left\Vert {{\bf{x}}}_{(UR{I}_{B})}\right\Vert }=\frac{\mathop{\sum }\limits_{i=1}^{n=100}{x}_{(UR{I}_{A})i}\ast {x}_{(UR{I}_{B})i}}{\sqrt{\mathop{\sum }\limits_{i=1}^{n=100}{x}_{(UR{I}_{A})i}^{2}}\sqrt{\mathop{\sum }\limits_{i=1}^{n=100}{x}_{(UR{I}_{B})i}^{2}}}$$

### Creating a similarity network

A similarity network is created where the different schema element URIs represent the nodes of this network. An edge exists between two nodes if: (*i)* the cosine similarity score between the corresponding URIs is greater than the threshold of 0.75, and (*ii)* there exists a one-to-one mapping between the two schema elements for the particular combination of LOD sources.

For example, consider three URI nodes *n*_1_, *n*_2_, *n*_3_, such that, *n*_1_, *n*_2_ ∈ ${\mathcal{L}}{\mathcal{D}}$_1_ and *n*_3_ ∈ ${\mathcal{L}}{\mathcal{D}}$_2_. If *Sim*(*n*_1_, *n*_2_) = 0.87, *Sim*(*n*_1_, *n*_3_) = 0.93 then an edge will only be created between *n*_2_ and *n*_3_ nodes. This criteria has an underlying assumption that there should exist a unique alignment between similar schema elements in different LOD sources. While the cosine similarity scores in the previous step can, by itself, aid in detection of similar schema elements (e.g., all schema elements pertaining to *Molecular weight*), this similarity network will aid in the detection of sources that contain relevant information pertaining to a given biomedical concept or relation type.

### Community detection using Louvain method

Finally, the Louvain method^72^ for community detection in weighted networks is used. The cosine similarity scores form the edge weights in the similarity network. This method has been used to detect communities of users using social networks and mobile phone usage data, as well as detect species in network-based dynamic models^73–75^. The Louvain method maximizes a ‘modularity’ statistic *Q*, which measures the density of edges inside communities compared to edges between communities. In the equation below (reproduced from Blondel, *et al*.^72^), 2*m* is the sum of all edge weights in the graph (i.e., $m=\frac{1}{2}{\sum }_{ij}\,Sim(i,j)$) and *k*_*i*_ is the sum of all weights to edges attached to node *i* (i.e. ${k}_{i}={\sum }_{j}\,Sim(i,j)$). $\delta ({c}_{i},{c}_{j})$ is a delta function that looks at the community assignments (*c*_*i*_ and *c*_*j*_) for connected nodes *i* and *j*.

4 $$Q=\frac{1}{2m}\sum _{ij}\left[Sim(i,j)-\frac{{k}_{i}{k}_{j}}{2m}\right]\delta ({c}_{i},{c}_{j})$$

5 $$\delta ({c}_{i},{c}_{j})=\left\{\begin{array}{ll}1 & if\,({c}_{i}={c}_{j})\\ 0 & if\,({c}_{i}\ne {c}_{j})\end{array}\right.$$

The communities are visualized using the Cytoscape platform^76^.

## Results

### LSLOD schema graph $\boldsymbol{\mathcal{G}}$_*LSLOD*_

Using the Extraction Algorithm, we extracted 99 RDF graphs from ≈20 SPARQL endpoints. The total number of classes, object properties, data properties, and datatypes extracted and linked in the ${\mathcal{G}}$_*LSLOD*_ schema graph were 57,748, 4,397, 8,447 and 24 respectively. The extractors can function on any SPARQL endpoint irrespective of its (*i)* version, (*ii)* support for different SPARQL keywords^39^, and (*iii)* the size of the underlying RDF graphs. The Extraction Algorithm can generally process most SPARQL endpoints within 4–8 hours. A visualization of a portion of the extracted ${\mathcal{G}}$_*LSLOD*_ schema graph is shown in Fig. 2b. A snippet of the extracted information for a given source is shown in Box 2.

Through an empirical analysis, we have found some overlap between the different schema elements, based on the way linked data publishers model the underlying data (i.e., some object properties in a given source are used as data properties in another LOD source). We found standard datatypes defined under the XML Schema Definition (XSD)^77^ and RDFS^36^ such as xsd:string, xsd:integer, xsd:float, xsd:dateTime, xsd:boolean, rdfs:Literal. The analysis also found an ontology class UO:0000034 (Units Ontology^78^ Week class) used as a datatype. In some cases, we also found that RDF graphs may exhibit semantic mismatch where instances are aligned to classes from an exhaustive ontology, such as ChEBI^6^ or NCIT^15^ that have more than 50,000 classes, using the rdf:type property. *Semantic Mismatch* in LOD is a form of semantic heterogeneity. Since rdf:type is a predicate in almost all the SPARQL query templates used by the Extraction Algorithm, semantic mismatch significantly increases the number of classes in the ${{\mathcal{G}}}_{{\mathcal{T}}}$_–*Box*_, and consequently the number of queries that need to be formulated for each class significantly increases.

### Vocabulary reuse across biomedical linked open data sources

We analyzed the 70,592 total schema elements (classes, object properties, and data properties) extracted from the LOD sources in ${\mathcal{G}}$_*LSLOD*_ for vocabulary reuse. In this research, we looked only at reuse of schema elements from vocabularies published at the Linked Open Vocabularies (LOV) repository^45^ and ontologies that are either published at the BioPortal repository^46^ or on the Web as OWL files available for download (and have .owl in the namespace prefix, for easy search).

**Fig. 4:**
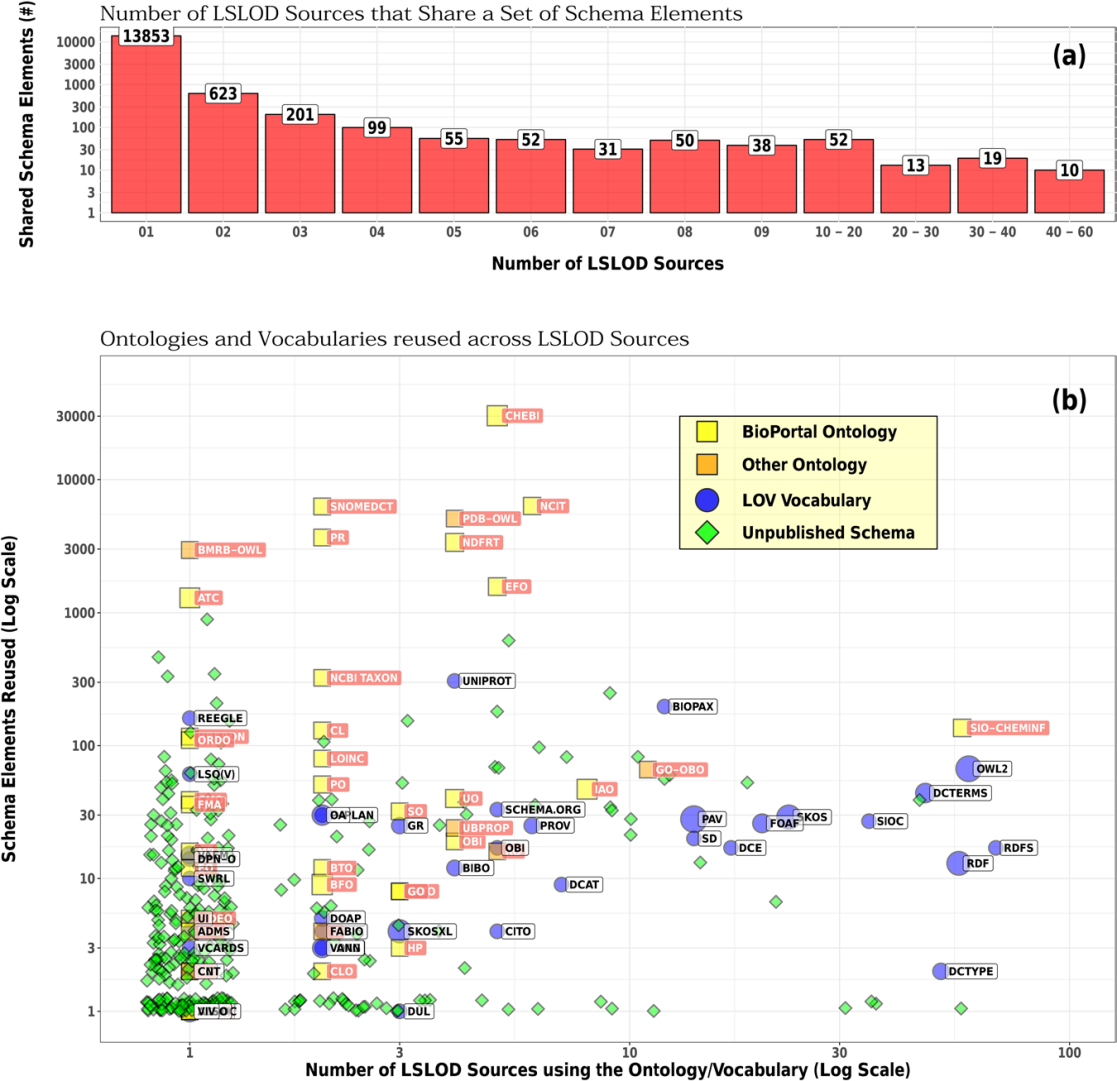
Vocabulary reuse across biomedical linked open data sources (RDF graphs): (**a)** A histogram of schema elements that are shared across multiple LOD Sources. It can be seen that most LOD Sources use a unique schema or vocabulary whose elements are not reused across any other data source. However, there are at least 100 schema elements that are shared by >10 LOD Sources. (**b)** The different vocabularies (published at LOV repository) and ontologies (BioPortal and other OWL ontologies) whose elements are reused in the schemas of LOD sources. Several LOD sources either use unpublished schemas or use elements from common vocabularies (e.g. DCTerms and SKOS).

Figure 4 shows the results of the analyses. Figure 4a depicts a histogram of schema elements that are shared across *X* number of LOD sources. It can be observed that most LOD sources are composed of unique schema elements that are present only in that particular source (i.e., the first histogram bar from the left). However, there are at least >100 schema elements that are shared between >10 LOD sources (i.e., the last four histograms from the right).

Terms are reused in biomedical LOD graphs from 34 BioPortal ontologies, 7 other biomedical ontologies, available on the Web but not stored in the BioPortal repository, and 43 LOV vocabularies (Fig. 4b). Some of the reused ontologies and vocabularies are very popular in biomedicine (e.g., ChEBI^6^, SNOMED CT^62^, NCIT — National Cancer Institute Thesaurus^15^, GO — Gene Ontology^19^) and in the Semantic Web community (e.g., DCTerms Metadata Description Model^79^, RDFS^36^, SKOS — Simple Knowledge Organization System^64^, Data Catalog Vocabulary^80^) respectively.

However, most of these biomedical ontologies are used only in the schemas of a few LOD sources (X-axis), and moreover only a few elements from these ontologies are reused (Y-axis). For example, the Anatomical Therapeutic Chemical Classification terminology^81^ — popularly used to classify drugs according to their mechanism of action — is used in the schema of only one LOD source. It can be seen that several LOD sources use unpublished schemas (green diamonds), hence, a large number of unique schema elements are present in only one source in Fig. 4a.

Several LOD sources do reuse schema elements from a set of widely used vocabularies (≈100 as per Fig. 4a). However, the elements of these vocabularies are not useful for data integration and integrated querying from a biomedicine perspective. It should be noted that several elements from the combination of the Semantic Science Ontology and Chemical Information Ontology (SIO-CHEMINF)^47,82^ are reused across several LOD sources and may be a popular resource for the emergence of a common data model for integrated querying of these sources. A large number of these LOD sources however only use one particular element from SIO-CHEMINF ontology..

Using Eq. 1, vocabulary reuse was determined to be 19.86%, which may indicate favorable reuse across LOD sources in comparison to ontologies. However, it should be noted that this statistic may be driven by the reuse of 30,251 ChEBI classes in a few sources. We also determined several cases of *semantic mismatch* where classes from popular biomedical ontologies in the BioPortal repository (e.g., SNOMED CT, NCIT, GO) are mapped to instances in these LOD sources.

**Fig. 5:**
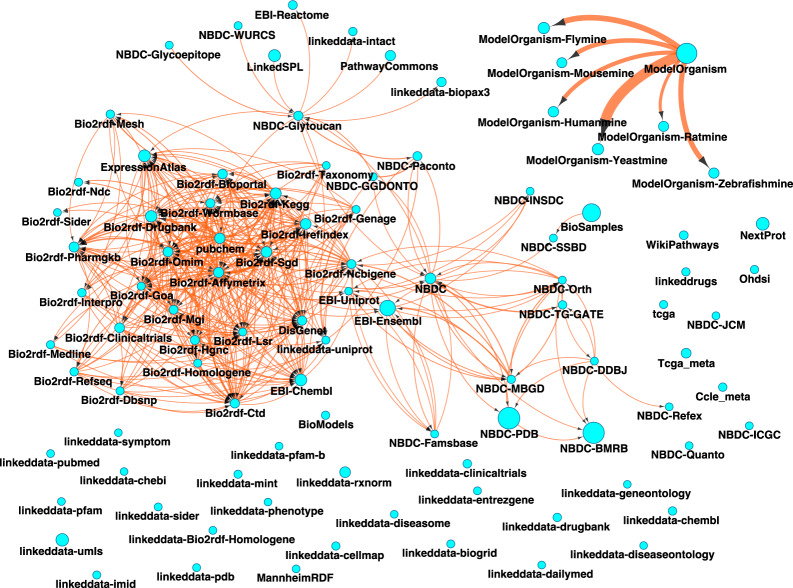
Inter-linking and intra-linking across biomedical linked open data sources (RDF graphs): Each unique RDF Graph retrieved by the Extraction Algorithm is shown as a node in this network. The size of the node indicates the number of unique object properties used to link entities in different classes from the same source. The size of the edge indicates the number of unique object properties used to link entities in different classes in different sources.

Figure 5 shows the different RDF graphs extracted as nodes in a network. The size of the node indicates the number of unique object properties used to link entities in different classes from the same source (intra-linking). The size of the edge connecting two different nodes indicates the number of unique object properties used to link entities in different classes in different sources or RDF Graphs (represented using the corresponding node). This network diagram can be perceived to be similar to the LSLOD cloud diagram^23^ (available at http://lod-cloud.net).

It can be observed that the LSLOD cloud is not actually as densely connected as is often indicated through the LSLOD cloud diagram. Several sources, which may exhibit a higher degree of intra-linking are not inter-linked with other RDF graphs and exist as stand-alone data sources converted using RDF. It can also be observed that the PDB RDF graph, exposed by the NBDC project, and the UniProt RDF graph, published by the EBI-RDF consortium, show a lack of connectivity. While the underlying data sources are similar and consist of information on proteins, the lack of connectivity can be explained if the NBDC project uses a different representation scheme for URIs used to denote protein entities as compared to the EBI-RDF consortium. The highly appreciated efforts by the Bio2RDF^20^ and EBI-RDF^60^ consortia can be seen in the dense inter-linking between the consortia-published sources.

### Communities of related content in the LSLOD cloud

While the low level of vocabulary reuse across LOD sources present a bleak picture of the Semantic Web vision toward facilitating Web-scale computation and integrated querying, it should be noted that there is significant similarity of content across these sources.

Using the pre-trained word embedding vectors and IDF statistics, we generated 100-dimensional URI embedding vectors for different schema elements URIs in the ${\mathcal{G}}$_*LSLOD*_ schema graph. Whereas the word tokens for most URI labels were found in the vocabulary, 3,340 word tokens, such as ‘differn’, ‘heteronucl’, and ‘neoplas’ were not found either due to typos or due to minimal occurrence frequency in publications. We represented these words using the default embedding vector and IDF statistic. We computed cosine similarity scores to detect additional mappings between different schema elements. For example, the drugbank:Molecular-Weight class has higher cosine similarity scores to related schema elements, such as kegg:mol_weight, biopax:molecularWeight, and MOLD:hasMolecularWeight data properties, as well as the CHEMINF:000198 (Molecular Weight Calculated by Pipeline Pilot) class.

A similarity network was created with 12,613 schema elements as nodes in this network. This number of nodes is less than the total number of schema elements in the ${\mathcal{G}}$_*LSLOD*_ schema graph (70,587). We excluded domain-specific classes that were reused from ChEBI and other ontologies through semantic mismatch. We also excluded URIs for which labels could not be extracted.

Edges were created between these nodes using the cosine similarity scores between the corresponding schema elements. There were 169,005 pairs of schema element URIs that satisfied the first criterion of having a cosine similarity value above the threshold of 0.75. This threshold was predetermined after an exploratory analysis of pairs of schema elements that satisfied this criterion. While it would make sense to have a higher threshold (≈0.95) for a higher degree of certainty on similarity, having a threshold of 0.75 allowed the inclusion of pairs, such as obo:regulates ↔ drugbank:mechanism-of-action: 0.75, chembl:mechanismDescription ↔ drugbank:mechanism-of-action: 0.85, faldo:location ↔ drugbank:cellular-location: 0.89. After applying the second criterion of one-to-one mapping between two schema elements for a particular combination of LOD sources, the network had 22,855 pairs of schema-element URIs for which edges were created.

**Fig. 6:**
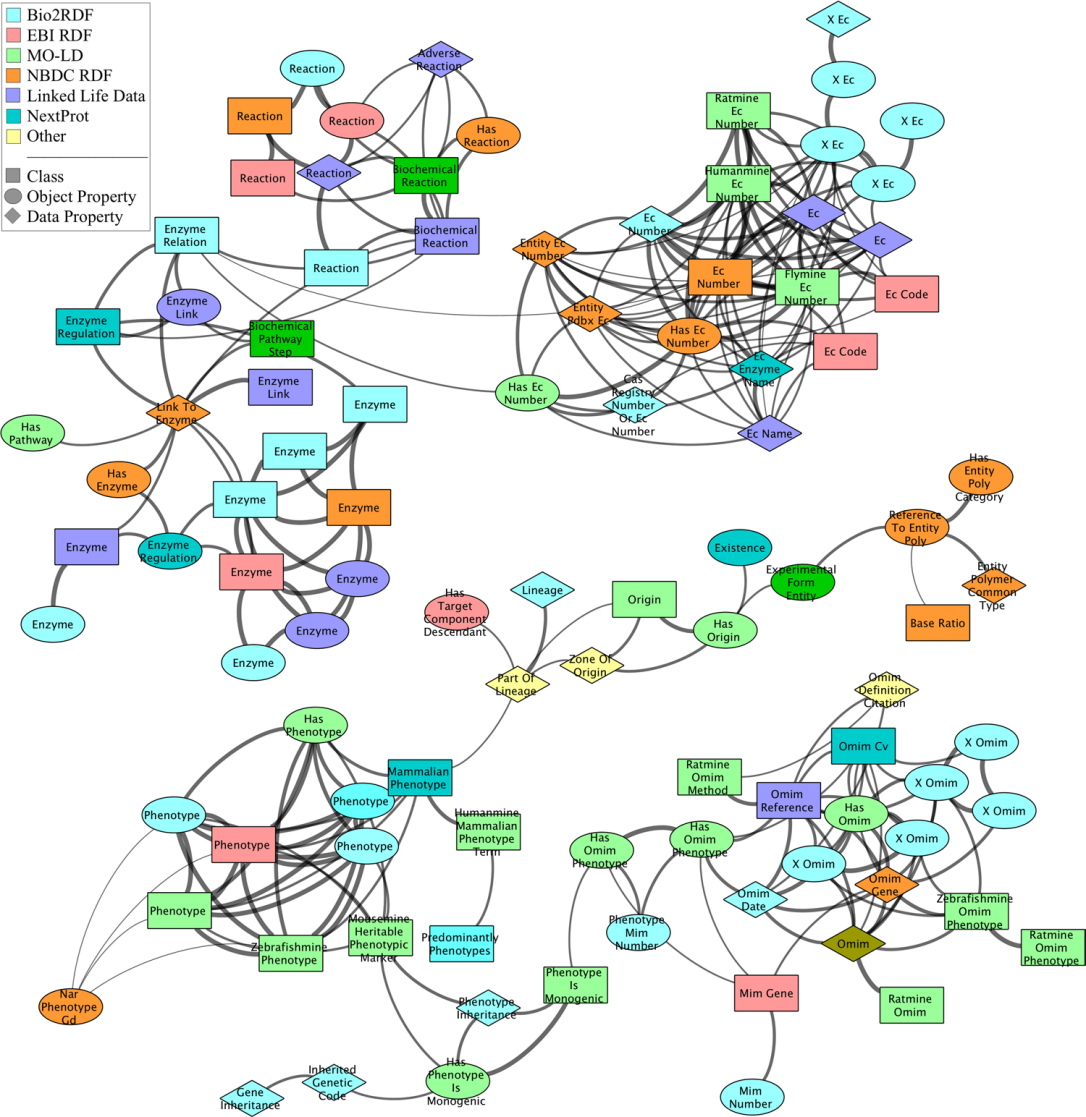
Communities of similar content across biomedical linked open data: Two communities composed of schema elements related to Enzyme and Phenotype concepts are visualized in a greater detail. The nodes indicate a distinct schema element (distinct URI), and are colored according to the LOD project they are present in. The shape of the nodes indicates the type of schema element (class, object property, data property). The width of an edge between two nodes is proportional to the cosine similarity score between the URI embeddings of the corresponding schema element. Smaller sub-communities related to Reaction, *Enzyme Commission Number* and *OMIM Phenotype Identifier* (Online Mendelian Inheritance in Man) are also observed.

We used the Louvain method for detecting communities in the weighted similarity network. The method terminated after achieving a maximum modularity statistic (i.e., the density of edges inside communities compared to edges between communities) of 0.817296. We detected 6,641 communities. While most of these communities had minimal membership (i.e., ≤10 schema elements were members), there were 51 communities with more than 10 schema elements as members. On visual inspection using the Cytoscape network visualization platform, we determined that these communities primarily consisted of schema elements related to a few key biomedical concepts or relation types. These communities are partially visualized in Fig. 3, with two communities composed of schema elements related to Enzyme and Phenotype concepts visualized in a greater detail in Fig. 6. Enzyme entities are specialized proteins which act as biological catalysts to accelerate biochemical reactions, whereas Phenotype entities are composite observable characteristics or traits of an organism.

In Fig. 6, each node is a distinct schema element URI (i.e., class, object property or data property) observed in the ${\mathcal{G}}$_*LSLOD*_ LSLOD schema graph. These nodes are colored based on the LOD project they are primarily present in (e.g., Bio2RDF, EBI-RDF). It should be noted that each LOD project (e.g., Bio2RDF) may publish multiple sources as distinct RDF graphs (e.g., KEGG^9^, DrugBank^11^). The shape of the nodes is reminiscent of the type of schema element — classes are represented as rectangular nodes, object properties as elliptical nodes, and data properties as diamond nodes. The nodes are labeled using the extracted labels, but the underlying URIs may be completely different. The width of the edge connecting different nodes is proportional to the cosine similarity scores between the URI embeddings of the connected schema elements.

It can easily be seen that different schema element URIs may be used across different sources (even if the sources are published by the same LOD project) to model and represent similar content and information (e.g., information on enzymes or phenotypes). It also showcases that data and knowledge relevant to a particular biomedical entity may be scattered across multiple sources (i.e., knowledge on a particular Enzyme entity can be retrieved from Bio2RDF, EBI-RDF and NextProt, and in many cases this knowledge may be novel). Other communities composed of schema elements focused on other key biomedical concept or relation types are also observed (e.g., Drug, Publication, Chromosome). The primary composition of a few of these larger communities (>30 members) is listed in Table 2. It should be noted that these communities may be composed of multiple sub-communities. For example, a sub-community of schema elements related to the Reaction concept is linked to the main cluster of nodes related to the Enzyme concept, since Enzyme entities are often associated with Reaction entities.

**Table 2:** Primary composition of communities in terms of key biomedical concepts.

Com. Id.	Total Elements	Key Biomedical Concepts
5023	549	**Clinical** (Age, Treatment, Summary, Dosage, Diagnosis)
		**Cancer** (Stage, Tumor, Cell, Sample, Rate)
		**Genomics** (Experiment, Target, Function, Interaction, Correlation, Assembly, Association, Expression, Genetic)
3098	281	**Protein and Genomic Sequences** (Sequence, Position, Start, Length, Protein, End, Repeat, Gene, Match, Begin, Translation, Exact, Exon, Frame, Distance)
2842	271	**Clinical** (Date, Condition, Entry, Process, Person, Diagnosis, Participant, Outcome, Indication, Therapy, Evidence, Access)
4346	260	**Data** (Code, Reference, Database, Sample, Data, Ontology, Semantic, Experiment, Dataset, Assembly, Registry)
2879	231	**Clinical** (Clinical, Group, ICD)
		**Genetic** (Gene, Entity, Ncbigene, Species, Atom, Ref)
3945	228	**Biomedical Relations** (Disease, Relationship, Expression, Gene, Level, Sample, Drug, Interaction, Component)
2515	211	**Protein** (Protein, Site, Region, Molecule, Domain, Binding, Interaction, Active, Atom, Canonical, Primary, Structure)
63676	210	**Literature** (Description, Author, Entity, Details, Note, Article, Issue, List, Type, Study, Entry, Document, Data, Address)
1417	192	**Identifiers** (Uniprot, Identifier, Accession, Ensembl, Taxonomy, PDB, Genbank, Gene, MGI, Database, Id, Refseq, NCBI, CDS)
1632	171	**Identifiers** (Id, Taxon, Mesh, Entity, Type)
		**Genomics** (Platform, Entry, Sample, Genus, Array, Gene, List)
3249	171	**Genotype–Phenotype Associations** (Gene, Symbol, Association, Product, Variant, Tag, Locus, Phenotype, Allele, Disease)
5652	158	**Genomics** (RNA, Probe, Strand, Direction, Region, DNA)
1446	132	**Software** (Version, Software, File, Format, Image, Email, Scale)
2535	126	**Drug** (Drug, Chemical, Action, Ingredient, Bond, Activity)
2421	110	**Chromosome** (Location, Chromosome, Entity, Cellular)
2317	105	**Pathway** (Pathway, Molecular, Formula)
		**Unit** (Weight, Body, Units, Volume, Unit)
1806	101	**Source** (Source, Natural, Vector, Derived, Tissue)
2672	99	**Literature** (Count, Text, Comment, Editor, List, Data, Notes)
6316	97	**Ontology** (Annotation, Ontology, Type, Structure)
5969	83	**Social** (Status, Gender, Authority, Interaction)
2429	79	**mRNA** (Transcript, mRNA, Signal, Frequency, Expression)
1868	77	**Literature** (Citation, Publication, Journal, Literature)
1349	67	**Assay** (Method, Assay, Type, Measurement)
6241	62	**Genetic** (Strain, Allele, Mutation, Genotype, SNP)
6052*	50	**Enzyme** (EC, Enzyme, Reaction)
3524*	50	**Phenotype** (Phenotype, Omim)
2490	50	**Organization** (Component, Complex, Assembly, Entity)
1849	49	**Interaction** (Interaction, Experiment, Strength)
2897	49	**Cell Line** (Cell, Line, Tissue, Type, Node)
6283	39	**Organism** (Organism, Host, Entity)
2911	38	**Ontology** (Synonym, Term, Ontology)
1787	32	**Evolution** (Member, Family, Orthologue, Homologue, Evidence)
5695	31	**Literature** (Pubmed, Keyword)

(*) marked communities are visualized in Fig. 6.

Smaller communities (<10 members) also consist of specific schema elements in different sources that are similar to each other but that are in label mismatch through use of different URIs (e.g., snoRNA is represented using

MOLD:yeastmine_SnoRNAGene, MOLD:humanmine_SnoRNA, EBIRDF:ensembl/snoRNA, BIO2RDF:ncbigene_vocabulary/SnoRNA-Gene, NBDC:mbgd#snoRNA). Knowledge of such communities will aid biomedical researchers to determine which sources in the LSLOD cloud contain information relevant to their domain of interest (e.g., research on small nucleolar RNAs snoRNA, a class of small RNA molecules that primarily guide chemical modifications).

## Discussion

Semantic heterogeneity across different data and knowledge sources, either in the use of varying schemas or entity notations, severely hinders the retrieval and integration of relevant data and knowledge for use in downstream biomedical applications (e.g., drug discovery, pharmacovigilance, question answering). Enhancing the quality and decreasing the heterogeneity of biomedical data and knowledge sources will lead to enhanced inter-disciplinary biomedical research, which will lead to developing better clinical outcomes and increasing our knowledge on biological mechanisms.

So far, significant resources have been invested to represent, publish, and link biomedical data and knowledge on the Web using Semantic Web technologies to create the Life Sciences Linked Open Data (LSLOD) cloud. However, there are very few biomedical applications that query and use *multiple* LOD sources and biomedical ontologies directly on the Web to generate new insights, or to discover novel implicit associations *serendipitously*. This is, in part, due to the semantic heterogeneity emanating from underlying sources, which has also penetrated the LSLOD cloud in varying forms. Some of the primary benefits of the LSLOD cloud — querying, retrieval, and integration of heterogeneous data and knowledge from multiple sources on the Web seamlessly — cannot be availed.

Linked data publishers are strongly encouraged to reuse existing models and vocabularies and to provide links to other sources in the LSLOD cloud^21,41^. Similarly, biomedical ontology developers are strongly encouraged to reuse equivalent classes existing in other ontologies, while building new ontologies^83,84^. There are several benefits if biomedical ontologies and LOD sources reuse content from existing sources — (*i)* reduction in data and knowledge engineering and publishing costs, (*ii)* decrease in semantic heterogeneity and increase in semantic interoperability across datasets, and (*iii)* ease of querying, retrieval, and integration of data from multiple sources simultaneously through existing query federation methods^48^. On the other hand, lack of reuse will manifest as increased semantic heterogeneity across different LSLOD sources.

It can be asserted through the findings presented in this research that the Life Sciences “Linked” Open Data cloud is not actually as densely connected as is often indicated through the ubiquitously-referenced LSLOD cloud diagram. Several sources, which may exhibit a higher degree of intra-linking are not inter-linked with other RDF graphs and exist as stand-alone data sources converted using RDF (Fig. 5). Similarly, Fig. 4 shows that several biomedical LOD sources use unpublished schemas. Biomedical LOD sources do reuse schema elements from a set of popular vocabularies. However, these vocabularies originate from the Semantic Web community (e.g., those that are mainly used to refer to data elements and attributes) rather than from the biomedical community (e.g., those that are used to provide normalization schemes for biomedical entities). Hence, the elements of these vocabularies are not useful for data integration and integrated querying from a biomedical perspective (e.g., for retrieving all drug–protein target interactions).

### Similar content across the LSLOD cloud

As seen in Fig. 6, several online sources have data and knowledge pertaining to Enzyme or Phenotype entities but use completely different schema elements. Communities of similar content depicted in Fig. 6, while showcasing the different schema element URIs to represent similar content, may also provide an idea on the different equivalence attribute URIs used to link entities across different sources to a specific entity coding scheme. For example, the Enzyme entities in the different sources of the LSLOD cloud are linked to the Enzyme Commission number (a numerical classification scheme for enzymes) using different attribute URIs with labels such as Ec Code, Ec Number, X Ec, and Has Ec Number. Similarly, Phenotype entities are linked to the OMIM phenotype identifiers (i.e., Online Mendelian Inheritance in Man is a knowledge base of human genes and genetic phenotypes^85^) using different attribute URIs with labels such as Omim Reference, X Omim, and Has Omim Phenotype. Communities that are composed entirely of different attribute URIs referring entities in different sources to a uniform coding scheme are also observed (e.g., Ensembl and HGNC Gene Coding scheme attributes). The knowledge on the different equivalence attribute URIs could guide query federation methods to perform entity reconciliation after the retrieval of information from multiple sources.

Through a manual inspection of such “identifier” communities (e.g., communities 1417 and 1632 in Table 2), we were able to detect different URI representations for similar entities (classes and instances). A few examples of different URI representations are shown in Table 3 for chemical entities of ChEBI^6^, PubChem^5^ and MeSH^86^ sources. Most LOD querying architectures rely on the existence of exact URIs to query multiple sources simultaneously (using data warehousing, link traversal or query federation methods)^13,17,40^. However, since these URIs are essentially different, querying architectures will not be able to integrate data and knowledge from different sources (e.g., protein–ligand binding information from PDB and biological assay experimental data from PubChem). These examples qualify as “intent for reuse” and add to the challenges of semantic heterogeneity across the LSLOD cloud.

**Table 3:** Different kinds of URI representations for chemical compounds observed in LOD Sources.

Entity Origin	URI Representation	Sources
**ChEBI**	http://purl.obolibrary.org/obo/CHEBI/*	LinkedSPL, PubChem, NBDC
		ExpresssionAtlas, BioSamples
	http://identifiers.org/chebi/CHEBI:	BioModels, PDB, WikiPathways
		Bio2RDF, PathwayCommons
	http://identifiers.org/obo.chebi/CHEBI:	BioModels, BioPax, WikiPathways
	http://www.ebi.ac.uk/chebi/	PDB, ChEMBL
	http://bio2rdf.org/chebi:	Bio2RDF
**PubChem**	http://identifiers.org/pubchem.compound/	Bio2RDF, WikiPathways, NBDC
**Compound**	http://rdf.ncbi.nlm.nih.gov/pubchem/compound/*	WikiPathways, PubChem
	http://bio2rdf.org/pubchem.compound:	Bio2RDF
	http://pubchem.ncbi.nlm.nih.gov/compound/	NBDC
**MeSH**	http://purl.bioontology.org/ontology/MESH/*	NBDC, PDB, LinkedSPL
	http://id.nlm.nih.gov/mesh/YEAR/*	MeSH, DisGenet, PDB
	http://identifiers.org/mesh/	Bio2RDF, DisGenet, PDB
	http://www.ncbi.nlm.nih.gov/mesh/	BioSamples
	http://rdf.imim.es/rh-mesh.owl#	DisGenet
	http://bio2rdf.org/mesh_resource:	Bio2RDF
	http://bio2rdf.org/mesh:	Bio2RDF

(*) marks the recommended representation(s). **Acronyms**: (NBDC) National Bioscience Database Center, (PDB) Protein Data Bank, (MeSH) Medical Subject Headings.

### Intent for reuse across the LSLOD cloud

A few LOD sources do reuse content from existing biomedical ontologies, but in many cases the reuse is limited to very few classes or involve incorrect representations. Previously, we have documented similar cases to be “intent for reuse” on the part of ontology engineers in the biomedical domains^48^. Table 3 documents similar cases of intent for reuse that were observed across the biomedical LOD sources. Essentially, these URI patterns generally have the same identifier and source ontology or vocabulary, but are reused from different versions of the source ontology or vocabulary, or represented using different notations or namespaces. These patterns cannot be considered as actual reuse, as these are different, and often incorrect, representations for the same terms, and no explicit mappings are found. Hence, the advantages of reuse cannot be experienced. By using the correct representations, the semantic heterogeneity (i.e., content overlap) can be reduced substantially. Ideally, the representation of the URI used at the “authoritative source” (i.e., the source where the respective semantic resource is published and regularly maintained) is the standard representation that should be consistently reused across other LSLOD sources. Determining the “authoritative source” is out of the scope for this meta-analysis, but is a field of active research^50^. The recommended representations in Table 3 are based off the “authoritative source”. Ontology engineers and linked data publishers may lack sufficient guidelines and tools to identify authoritative sources for any semantic resource and use the recommended representation while reusing or referring to that semantic resource.

Intent for reuse and semantic heterogeneity severely hinders most query federation and link traversal methods for heterogeneous data and knowledge integration across the LSLOD cloud. For a more concrete example, consider the user query *“Retrieve Assays which identify potential Chemopreventive Agents that have Molecular Weight* < *300 g/mol and target Estrogen Receptor present in Human.”* This query requires the retrieval of knowledge from the KEGG knowledge base^9^ on all the chemicals that target Estrogen Receptor protein, and then the retrieval of assay data on these chemicals from the ChEMBL chemical bioactivity repository^59^. The KEGG knowledge base and the ChEMBL data repository are both published on the LSLOD cloud^20,60^, and have chemical URIs that have x-ref links to chemical URIs in the ChEBI ontology of biological chemicals^6^. However, since Bio2RDF KEGG uses the URI representation scheme http://bio2rdf.org/chebi:* and ChEMBL uses the URI representation scheme http://purl.obolibrary.org/obo/CHEBI/* for the same identifiers in the ChEBI ontology (Table 3), it is impossible to formulate a federated SPARQL query that can query KEGG and ChEMBL, simultaneously and retrieve the relevant results in an integrated fashion.

Increasing the reuse of schema elements from existing vocabularies and ontologies may decrease semantic heterogeneity and enable the integrated querying of multiple data and knowledge sources across the LSLOD cloud using the Semantic Web technologies. In cases where linked data publishers have the need to use a custom vocabulary or ontology, the overarching advice would be to publish this vocabulary or ontology to a popular repository, so other publishers can use these resources. The challenges for data and knowledge integration due to semantic heterogeneity across fragmented and incompatible data sources will also prevail as the wider data science community moves toward the adoption of the FAIR (Findable, Accessible, Interoperable, Reusable) principles^87^ for publishing and reusing open datasets on the Web.

Over the years, the Semantic Web community has recommended several best practices and standards on how to reuse schema elements from existing vocabularies and ontologies when publishing LOD sources^30,41,50,88^. However, centralized top–down approaches may be needed to evaluate and enforce the use of these standards and existing ontologies and vocabularies (e.g., SKOS^64^, Schema.org^65^, Semantic Science Ontology^82^) when a new source is published in the LOD cloud. Such centralized approaches are already being developed to monitor the accessibility and availability of the different sources in the LOD cloud^30,33,89,90^, and can be extended to avoid the proliferation of incompatible and redundant LOD sources and reduce our reliance on reuse standards and best practices that would emerge through an evolutionary process.

### Limitations and future work

The use of word embeddings in the community detection algorithm enables the identification of mappings between similar LOD schema elements (e.g., kegg:therapeutic-category ↔ drugbank:Drug-Classification-Category: 0.93, obo:regulates ↔ drugbank:mechanism-of-action: 0.75, faldo:location ↔ drugbank:cellular-location: 0.89). These mappings may provide suggestions to linked data publishers and consumers on relevant information in different sources. However, many of these mappings may not be accurate due to reliance on the word embeddings, as well as the 0.75 threshold on the cosine similarity scores. For example, as seen in Fig. 6, while the Reaction concept in the Enzyme community symbolizes biochemical reactions where enzyme entities may act as catalyzing agents, an error with the community detection algorithm also indicates that Adverse Reaction (clinical concept observed in patients treated with a set of drugs) may be a part of this community. This is due to the fact that the singular schema element pertaining to Adverse Reaction from an RDF graph in the Linked Life Data project^91^ is linked to schema elements pertaining to Biochemical Reaction (i.e., the edges have a cosine similarity score of more than 0.75).

In this research, we have limited this meta-analysis to only include the BioPortal repository^46^ and Linked Open Vocabularies (LOV) catalog^45^ as background sources of biomedical ontologies and LOD vocabularies. However, several other repositories may contain ontologies and vocabularies which are useful toward this meta-analysis (e.g., Ontology Lookup Service – OLS^92^). There were two underlying assumptions behind this decision: *i)* There may be very few biomedical ontologies and vocabularies that are not present in BioPortal and LOV catalog, but are exposed in another public source (e.g., As of October 2020, OLS has 252 ontologies whereas BioPortal has 896 ontologies, and only around 40 ontologies in OLS are not present in BioPortal. Out of these 40 ontologies, it should also be noted that 30 ontologies are “organism” ontologies which may have domain-specific concepts not present in the LSLOD cloud), and *ii)* the LSLOD cloud diagram at https://lod-cloud.net indicates that LOD sources are more intricately linked to biomedical ontologies in BioPortal repository. This meta-analysis can be extended in the future to include other additional sources of background knowledge.

While we have tried to conduct the meta-analysis by classifying the LSLOD sources according to the project that publishes them (e.g., Bio2RDF, EBI, NBDC), a further analysis can be conducted in conjunction with author citation networks to discern the common publishers across different LSLOD sources (e.g., publishers of Bio2RDF^20^ sources may be involved with the publishing efforts of WikiPathways^61^ or PubChem^5^ RDF graphs). This would enable us to determine the true independent publishers of LSLOD sources. Ideally, data publishing, curation, reusability, and governance should be a community-driven process, with monitors to ensure that the best practices and standards are followed by the different RDF sources.

For this research, the meta-analysis was conducted over the April 2018 snapshot of the LSLOD cloud, as well as using the biomedical ontologies and RDFS vocabularies from that time period. However, the LSLOD cloud is constantly evolving and the biomedical data and knowledge sources are published, updated, or deprecated on a regular basis. In the future, the methods presented in this research can be embedded within a Web application which automatically extracts schema elements from any novel biomedical data or knowledge source, which is registered by linked data publishers, and analyzes these elements in the context of the current LSLOD cloud and biomedical resources stored in public catalogs (e.g., BioPortal^46^ and the LOV catalog^45^). A formatted report with analyses results and recommendations can be issued to the data publishers empowering them to improve reuse in their source. This Web application, while outside the scope of the current meta-analysis research, can also continually monitor the LSLOD cloud to generate insights on the evolution, availability, quality, and semantic heterogeneity, of the different sources in the LSLOD cloud on a regular basis.

## Conclusion

Semantic Web and linked data technologies garner significant interest and investment from the biomedical research community toward tackling the diverse challenges of heterogeneous biomedical data and knowledge integration. In this paper, we conduct an empirical meta-analysis to demonstrate that there is considerable heterogeneity and discrepancy in the quality of Life Sciences Linked Open Data (LSLOD) sources on the Web, which are published using the above technologies. We discuss on whether the LSLOD cloud can truly be considered *“linked”* due to the heterogeneous schemas, varying entity notations, the lack of mappings between similar entities, and the lack of reuse of common vocabularies. While increasing reuse across biomedical ontologies and LOD sources is definitely considered to be an attractive alternative to decrease semantic heterogeneity, it will take a while for biomedical ontology developers and linked data publishers to realize the incentives of reuse and actually embrace the guidelines and best practices while publishing data and knowledge. Actual reuse through the use of correct URI representations in biomedical ontologies and LOD sources, rather than “intent to reuse”, can decrease semantic heterogeneity substantially itself. The findings from our meta-analysis across the LSLOD cloud, as well as the resources such as the LSLOD schema graph made available through this research, will lead to the development of better tools and methods to increase vocabulary reuse and enable efficient querying and integration of multiple heterogeneous biomedical sources on the Web.

## Data Availability

The LSLOD Schema Graph extracted from the different SPARQL endpoints of the biomedical LOD graphs in the LSLOD cloud is made available in the JavaScript Object Notation (JSON) format serialized as a Pickle file in the figshare repository (10.6084/m9.figshare.12402425.v2) under a CC BY 4.0 license^93^. The project also contains tab-separated values files consisting of information about extracted classes, object properties, data properties, datatypes, as well as of statistics on the LOD graphs from the LSLOD cloud used in this research.

## Code availability

The different visualizations and observations presented in this research are also made available at the project website (http://onto-apps.stanford.edu/lslodminer). The scripts for the extraction algorithm and the community detection algorithm used in this research are made available on GitHub (https://github.com/maulikkamdar/LSLODQuery).
